# Multiscale X-ray phase-contrast CT unveils the evolution of bile infarct in obstructive biliary disease

**DOI:** 10.1038/s42003-024-06185-7

**Published:** 2024-04-23

**Authors:** Xiaohong Xin, Jianbo Jian, Xu Fan, Beining Qi, Yuanyuan Zhao, Wenjuan Lv, Yuqing Zhao, Xinyan Zhao, Chunhong Hu

**Affiliations:** 1 School of Biomedical Engineering and Technology, Tianjin Medical University, Tianjin, 300070 China; 2 Department of Radiation Oncology, Tianjin Medical University General Hospital, Tianjin, 300052 China; 3 Liver Research Center, Beijing Friendship Hospital, Capital Medical University, Beijing, 100050 China; 4 Beijing Key Laboratory of Translational Medicine on Liver Cirrhosis and National Clinical Research Center of Digestive Disease, Beijing, 100050 China

**Keywords:** Biliary tract disease, X-ray tomography, 3-D reconstruction

## Abstract

Bile infarct is a pivotal characteristic of obstructive biliary disease, but its evolution during the disease progression remains unclear. Our objective, therefore, is to explore morphological alterations of the bile infarct in the disease course by means of multiscale X-ray phase-contrast CT. Bile duct ligation is performed in mice to mimic the obstructive biliary disease. Intact liver lobes of the mice are scanned by phase-contrast CT at various resolution scales. Phase-contrast CT clearly presents three-dimensional (3D) images of the bile infarcts down to the submicron level with good correlation with histological images. The CT data illustrates that the infarct first appears on day 1 post-BDL, while a microchannel between the infarct and hepatic sinusoids is identified, the number of which increases with the disease progression. A 3D model of hepatic acinus is proposed, in which the infarct starts around the portal veins (zone I) and gradually progresses towards the central veins (zone III) during the disease process. Multiscale phase-contrast CT offers the comprehensive analysis of the evolutionary features of the bile infarct in obstructive biliary disease. During the course of the disease, the bile infarcts develop infarct-sinusoidal microchannels and gradually occupy the whole liver, promoting the disease progression.

## Abstract (web summary)

A phase-contrast CT study of obstructive biliary disease suggests that the evolution of bile infarct and the formation of infarct-sinusoidal microchannels promote the disease progression.

## Introduction

Bile infarct defined as fields of hepatocyte necrosis under a cholestatic state was first described in 1887 by Jean-Martin Charcot and Albert Gombault, also known as “Charcot-Gombault necrosis”^1^. It is one of the characteristic histopathological features of obstructive biliary disease, including biliary atresia in infants, choledocholithiasis and malignancies such as pancreatic head tumor and cholangiocarcinoma^2,3^, a group of serious diseases that can lead to biliary fibrosis or cirrhosis and even death if the obstruction is not removed or bypassed in time^4,5^. The bile infarct appears at the onset of obstructive biliary disease and plays an important role in the disease progression^6^; therefore, monitoring the morphological alterations of the infarct during the course of the disease, preferably in three-dimensional (3D) model, is an essential step in understanding the mechanisms of the disease evolution.

Histology is the gold standard for identifying the bile infarct and observing its microstructure at the cellular level^7–9^, but unfortunately the two-dimensional (2D) nature of the histology does not allow for a complete representation of the 3D structure of the infarct and an accurate quantification of its morphological changes^10,11^. In vivo fluorescence microscopy employing appropriate fluorescent tracers is currently a popular tool for exploring the bile infarct, and researchers have used the system to capture, in real time, the live events of infarct formation in the liver of the bile duct ligation (BDL) model^6,12^. Nevertheless, the major limitation of this imaging technique is the depth of detection, which mainly provides high-resolution images within the surface of the liver^13^ and cannot detect the evolution of infarcts within the whole liver. To address these limitations, an imaging tool that can not only capture the infarct structures at a near-histological level but also has the ability to image the intact liver is needed.

Phase-contrast computed tomography (CT) is an X-ray imaging modality in which the phase shift of X-rays passing through matter is used to generate tissue contrast^14^. Prior work has demonstrated that phase-contrast CT yields higher contrast in soft tissue than absorption-based X-ray imaging that employs X-ray attenuation as the image contrast^15,16^. Combined with synchrotron sources, which have high coherence and super brightness, phase-contrast CT can image soft tissue at micron- or even nanometer-scale resolution^17^. With the aid of this technique, the main structures, such as the microvasculature^18^, hepatic sinusoids^19^ and bile ducts^20^ in the liver, can be clearly distinguished on phase-contrast CT images at various scales. Thus, multiscale synchrotron radiation phase-contrast CT has great potential to comprehensively characterize the evolution of bile infarcts in obstructive biliary disease.

In this study, the livers of a BDL model, a frequently used model of obstructive cholestasis in rodents^21^, at different time points were imaged using a multiscale synchrotron radiation phase-contrast CT technique. Subsequently, the evolutionary pattern of bile infarcts and the key morphological mechanism of the infarcts promoting disease progression were revealed.

## Results

### Phase-contrast CT revealed bile infarcts in BDL mice, confirmed by corresponding histological findings

**Fig. 1:**
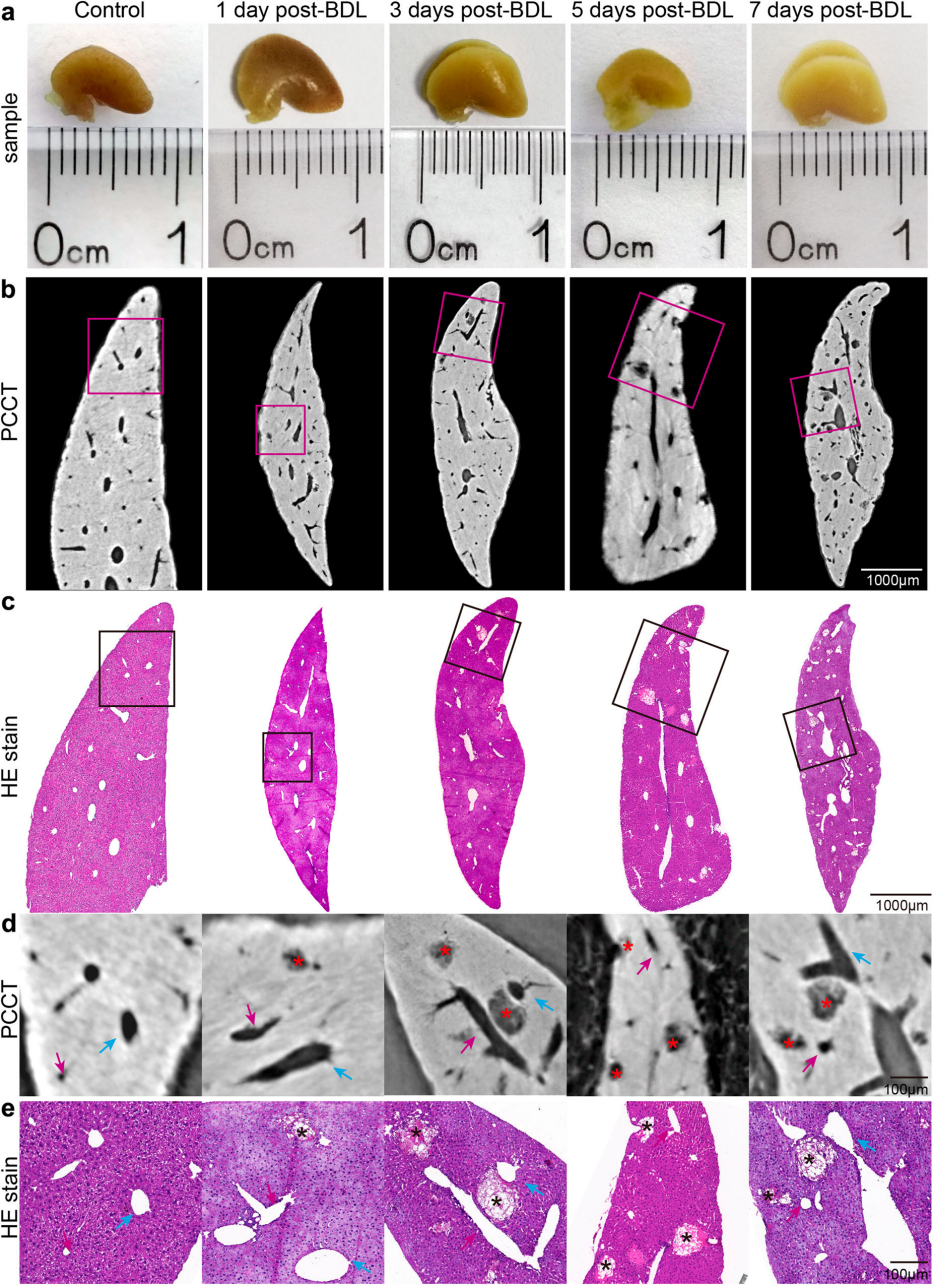
Comparison of phase-contrast CT slices with corresponding histological sections of liver lobes in mice at 1, 3, 5, and 7 days after BDL. **a** Photographs of intact liver lobes waiting for phase-contrast CT imaging (scale in millimeters). **b** Phase-contrast CT slices of the livers at 3.25 µm pixel size. **c** HE-stained histological sections corresponding to phase-contrast CT slices in (**b**). **d**, **e** Enlarged images of the regions of interest in (**b**) and (**c**). Asterisks indicate bile infarcts. The blue arrows represent the hepatic veins, and the portal veins are indicated by pink arrows.

Intact liver lobes in mice from different time points after BDL were imaged by phase-contrast CT with a pixel size of 3.25 µm (Fig. 1a). The CT slices clearly revealed the bile infarcts and vessels in the livers, and the smallest distinguishable infarct was approximately 10 µm in diameter (Fig. 1b). Additionally, pathological experiments were conducted on the imaged livers to validate the accuracy of phase-contrast CT in the imaging of bile infarcts (Fig. 1c). The results showed that the size, shape and distribution of the infarcts in the CT data were consistent with those in the histological findings, which was particularly clear on the enlarged images of the regions of interest (Fig. 1d, e).

### 3D visualization of the bile infarcts and vascular trees in BDL mice

**Fig. 2:**
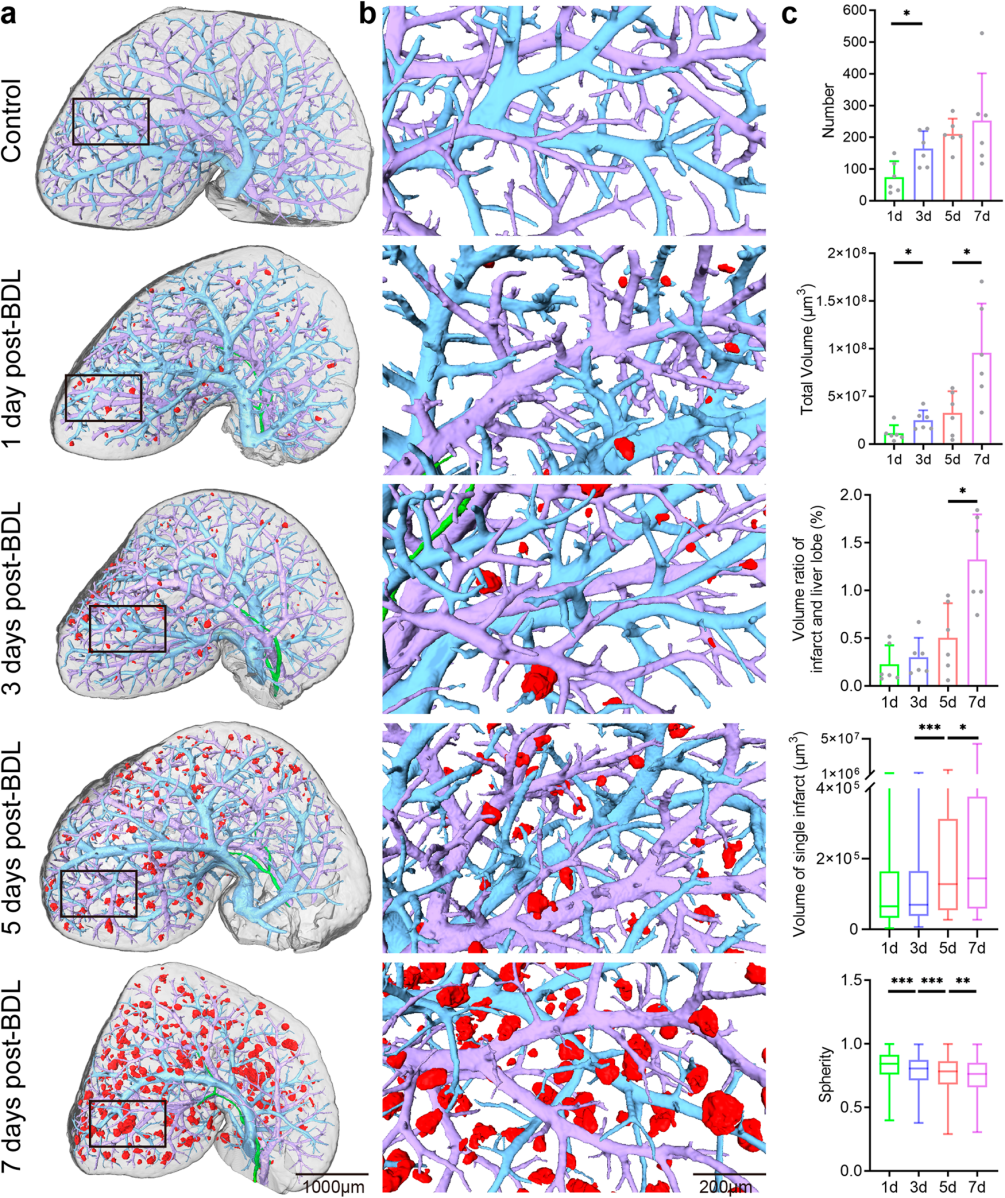
3D images reveal the distribution and quantitative information of the bile infarcts in mice after BDL. **a** 3D surface reconstruction of bile infarcts (red), hepatic vein (blue), portal vein (purple), and part of the bile duct (green) in intact liver lobes (gray). **b** Magnifications of rectangles in (**a**). **c** Bar graphs show the number of bile infarcts, total volume of bile infarcts and volume ratio of the bile infarcts in one liver lobe, displayed as means ± SD, and box plots show volume of a single bile infarct and sphericity of the bile infarct, with horizontal lines representing the maximum, upper quartile, median, lower quartile, and minimum values. *=*P* < 0.05, **=*P* < 0.01, ***=*P* < 0.001. The source data underlying this figure can be found in Supplementary Data 1.

Although CT slices could provide almost the same structural information as histology, the enormous potential of the phase-contrast CT method lay in 3D visualization and quantitative analysis, which had been demonstrated in Fig. 2. Based on 3D reconstruction technology, the 3D structures in intact livers from different time points of BDL, such as bile infarcts, vascular tree and bile ducts, were clearly shown in Fig. 2a. To further observe the association between the infarcts and the peripheral vascular tree, enlarged images were presented in Fig. 2b. The scenario in the 3D view of the infarcts was similar to variable-sized “mature fruits” (bile infarcts) hanging over the “branches” (portal vein or hepatic vein tree), and an increasing number of “mature fruits” turned out over time (days after BDL). In addition to 3D observation, quantitative analysis of the infarcts was also performed (Fig. 2c). The number of infarcts had close to a linear increase, consistent with the 3D images. The increase in the total volume of the infarcts was stronger, almost approaching exponential growth, and that of the 7-days group was 9 times that of the 1-day group. Correspondingly, their volume in the liver lobe showed the same trend as the total volume, implying that they occupied an increasing amount of space in the liver. For a single infarct, its volume growth was flatter from days 1 to 3 after BDL, with an obviously jump from day 5, after which the growth tended to flatten again. In contrast, the sphericity of the infarct was progressively reduced, indicating that their shape and surface tended to be irregular and uneven.

### Morphology and spatial distribution of the bile infarcts in the livers

In the 3D views, the bile infarcts were distributed disorderly in the livers, and there was no obvious pattern. Thus, 3D segmentation of the liver was performed to analyze the morphology and spatial distribution of the bile infarcts by the days post BDL.

**Fig. 3:**
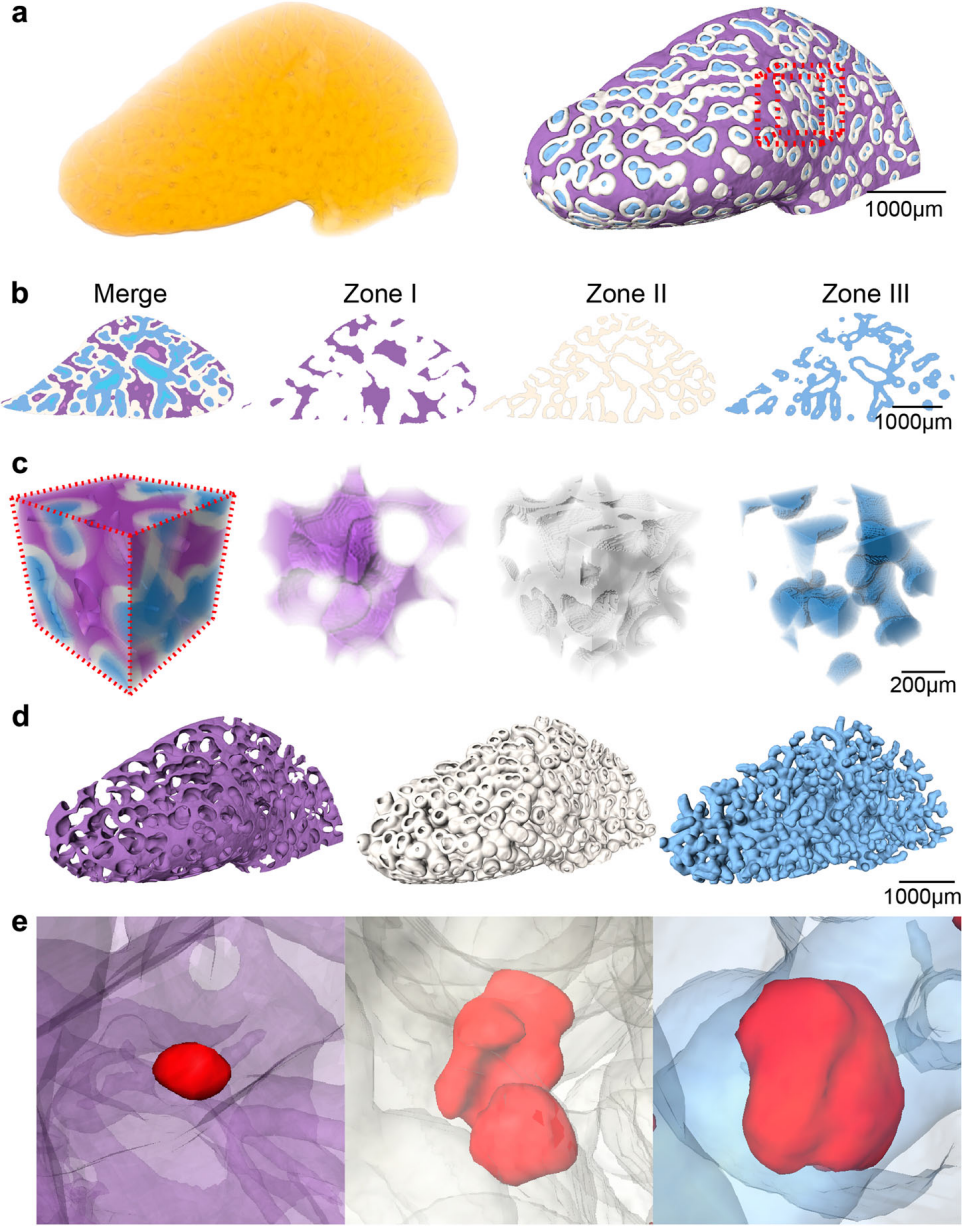
The proposed 3D hepatic acinus model. **a** Volume rendering of the liver (left); virtual division of the liver into zone I (purple), zone II (white), and zone III (blue) (right). **b** Areas of the three zones in the same slice. **c** The volume of interest (VOI) in (**a**). **d** Zone I, zone II, and zone III in a 3D modality. **e** The infarcts (red) are distributed in different zones.

The liver acinus extended from two dimensions into  three dimensions. The hepatic acinus in two dimensions was not suitable for analyzing the spatial distribution of the 3D bile infarcts, and thus, a 3D hepatic acinus model was proposed in the present study (Fig. 3). According to the 3D acinar model, the liver was equally divided into three zones (excluding the portal and hepatic/central vein regions), named zone I, zone II and zone III (Fig. 3a). The shape of each zone differed from the others in the same CT slice (Fig. 3b). A magnification of a cubic region of interest within the liver lobe showed the three zones in the 3D modality and the irregular shapes of the three zones on the slices (Fig. 3c). In the 3D modality, zone III had a tree-like structure, zone I had a wormhole-like structure, and zone II had features of both (Fig. 3d and see Supplementary Movie S1). The bile infarcts located in each zone were also presented (Fig. 3e).

**Fig. 4:**
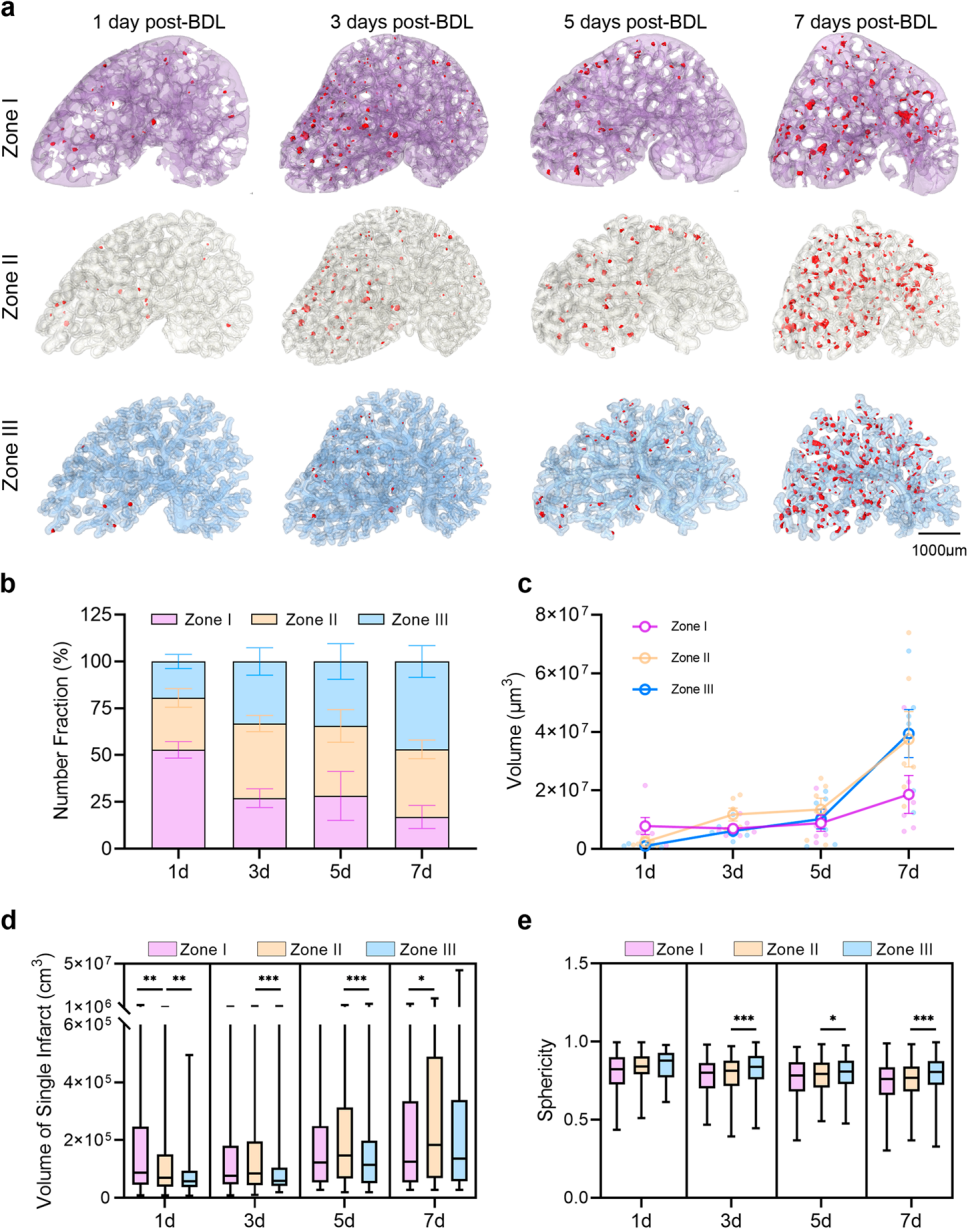
The bile infarcts in zone I, zone II, and zone III. **a** The distribution of bile infarcts (red) in the three areas. **b** The number fraction of the bile infarcts (means ± SD). **c** Total volume of the bile infarcts in each group (mean ± SEM). **d**, **e** The volume and sphericity of a single bile infarct in different zones, respectively. The horizontal lines of the box plots represent the maximum, upper quartile, median, lower quartile, and minimum values. * = *P* < 0.05, ** = *P* < 0.01, and *** = *P* < 0.001. The source data underlying this figure can be found in Supplementary Data 1.

Bile infarcts migrated from zone I to zone III in the 3D hepatic acinar model. The bile infarcts were investigated within zone I, zone II, and zone III after BDL (see Supplementary Movie S2). On day 1 post-BDL, the infarcts mainly appeared in zone I, and a few infarcts with a small volume were found in other zones (Fig. 4a). However, on the 7th day, the situation reversed. Although the number of infarcts in zone I also increased on day 7, the growth of zone II and zone III was more obvious. The results of quantitative analysis of the number of infarcts also confirmed this point. The number fraction of bile infarcts was more than half in zone I on day 1; however, it was reversed and less than half in zone III on day 7 (Fig. 4b). The total volume of the bile infarcts in zone I increased slowly, increased quickly in zone II, and increased sharply in zone III with time (Fig. 4c). The volume of the single infarct also increased with time in zone I, zone II, and zone III. The volume of the single bile infarct had a wide range in each subgroup, and the differences among the three zones were obvious (Fig. 4d). The sphericity of the infarcts in each zone was consistent with the overall data measured previously, with a gradual decrease after BDL; however, the data in zone III were noticeably higher than those in the other zones, indicating that the infarcts in this zone had the most regular morphology (close to spherical shape) (Fig. 4e).

### The bile infarcts grow individually or confluently

**Fig. 5:**
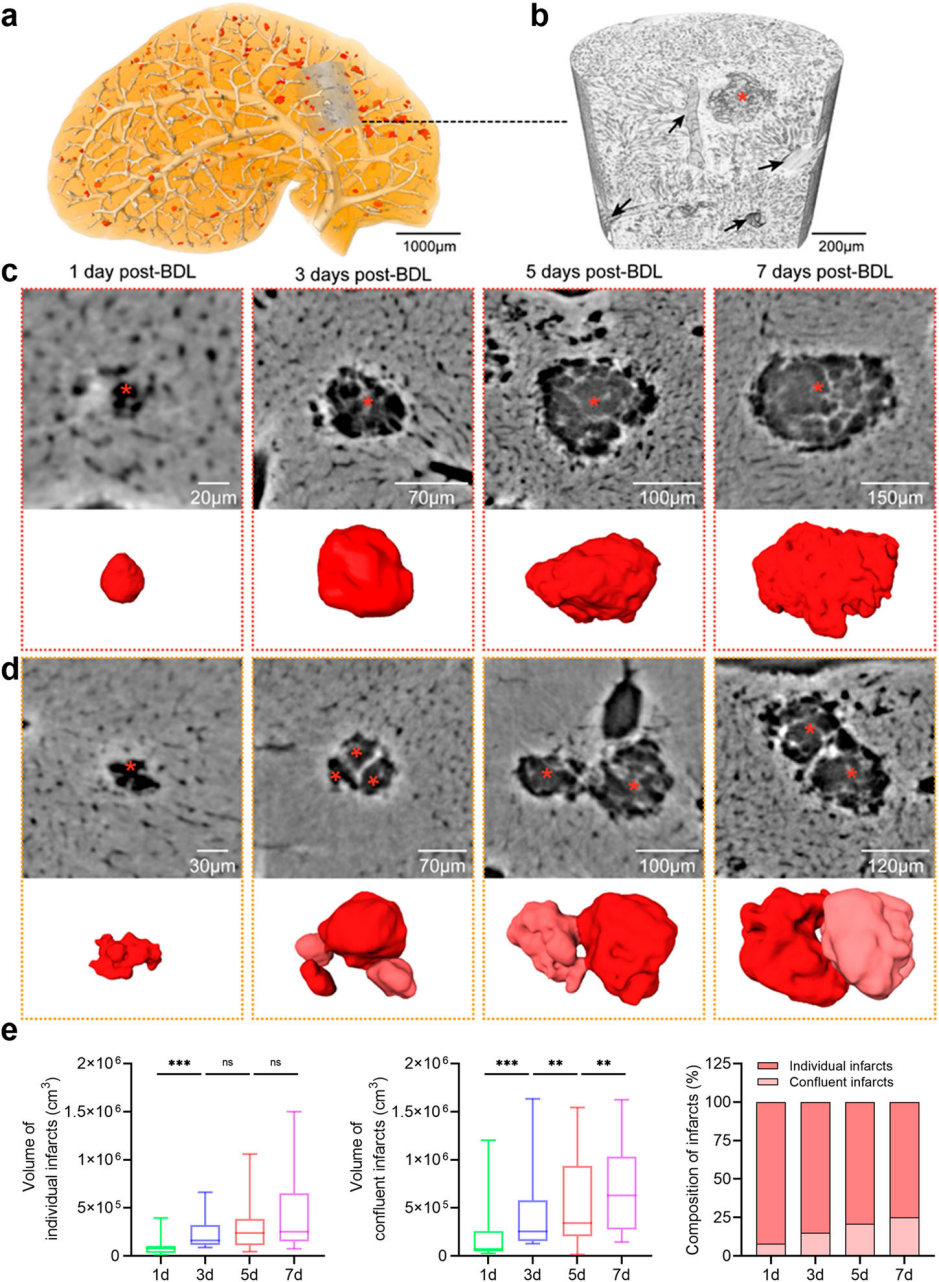
The infarcts grew in two forms: individually and confluently. **a** The volume rendering of the liver lobes reveals the vessels and the bile infarcts at 3.25 µm pixel size. **b** The volume rendering of the volume of the interest at 0.65 µm pixel size in (**a**). Asterisks indicate bile infarct; black arrows indicate vessels. **c** CT slices and 3D surface reconstruction of the individual bile infarcts. **d** CT slices and 3D surface reconstruction of the confluent bile infarcts. **e** The volume quantification of the individual bile infarcts and the confluent bile infarcts, and the composition of the infarcts in each group. The horizontal lines of the box plots represent the maximum, upper quartile, median, lower quartile, and minimum values. ** = *P* < 0.01, *** = *P* < 0.001 and ns = no significance. The source data underlying this figure can be found in Supplementary Data 1.

To determine the growth pattern of the bile infarcts, some volumes of interest (VOIs) were imaged with a higher resolution (Fig. 5a, b). At the submicron scale (0.65 µm), more details of the bile infarcts could be seen to analyze the process of the infarcts from small to large (Fig. 5c, d). From these details, two typical growth patterns of the infarcts were found. One was the continuous expansion of the individual infarcted area; that was, the microinfarct (necrosis of several hepatocytes) was formed first, and the surrounding hepatocytes gradually necrotized with the time after BDL to form a large infarct (Fig. 5c). The surface of independently growing infarcts changed from smooth to uneven, and the volume gradually increased after BDL. The other was that several infarcts gradually fused, and finally, part of the boundary disappeared to form a large infarct (Fig. 5d). From the beginning of BDL, the confluent infarct showed that several infarcts were connected. Later, the fusion phenomenon became increasingly obvious, and the volume also increased synchronously (Fig. 5e). The ratio of confluent bile infarcts in each group was 8.0%, 15%, 21%, and 25% (Fig. 5e).

### Identification of bile infarct-sinusoidal microchannels at the submicron scale

**Fig. 6:**
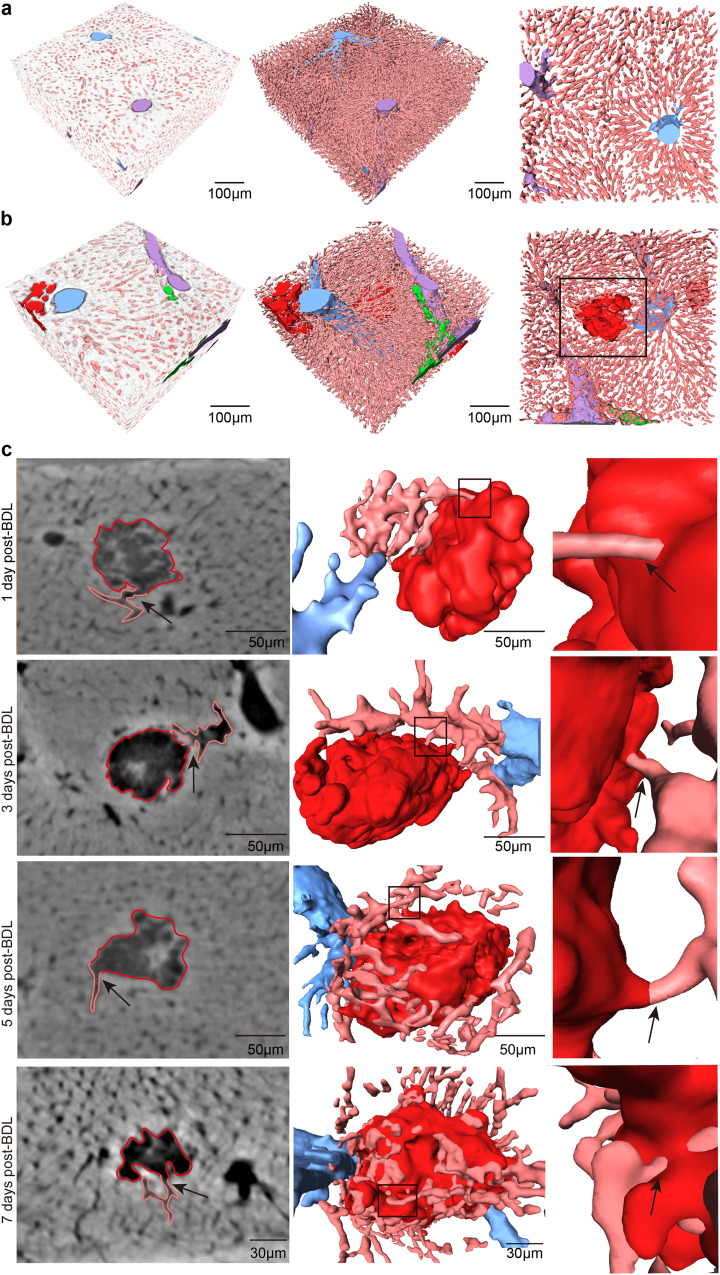
3D visualization of the microchannels between the bile infarcts and hepatic sinusoids at the submicron scale (0.65 µm). **a** High-resolution display of the hepatic lobule structure including liver parenchyma (white), interlobular portal vein (purple), central vein (blue) and hepatic sinusoids (pink) in the control group. **b** The bile infarct (red in black box) invaded and occupied the space of the sinusoids, connecting with the sinusoids at the boundary. **c** CT slice and 3D surface reconstruction of the connecting microchannels (black arrows) between the bile infarcts and sinusoids.

At the submicron level, phase contrast CT clearly revealed the spatial structure of the classical hepatic lobular architecture in the livers. In the normal hepatic lobule, the hepatic sinusoids and the hepatic plate composed of hepatocytes were arranged radially around the central vein (Fig. 6a), while in the BDL model, the bile ducts underwent marked dilation, and the bile infarcts invaded part of the liver parenchyma space, forming an irregular cavity structure in 3D (Fig. 6b). Moreover, by analyzing the border area between the hepatic sinusoid and infarct, it was found that there was a microchannel between them (see Supplementary Movie S3, in which virtual endoscope technology was used to show this microchannel). The microchannels around the infarcts from 1 day to 7 days after BDL were reconstructed (Fig. 6c). It was found that the existence of this microchannel was a common phenomenon. It began to appear from day 1, and the number of the microchannel in a single infarct showed a gradually increasing trend with increasing time (from day 1 to day 7: mean, 1.86 ± 0.87, 2.47 ± 1.28, 3.38 ± 1.79, 5.47 ± 2.87, *P* < 0.01, 25 infarcts were tested in each time class). The average diameter of this microchannel is 3.41 ± 0.26 µm.

## Discussion

**Fig. 7:**
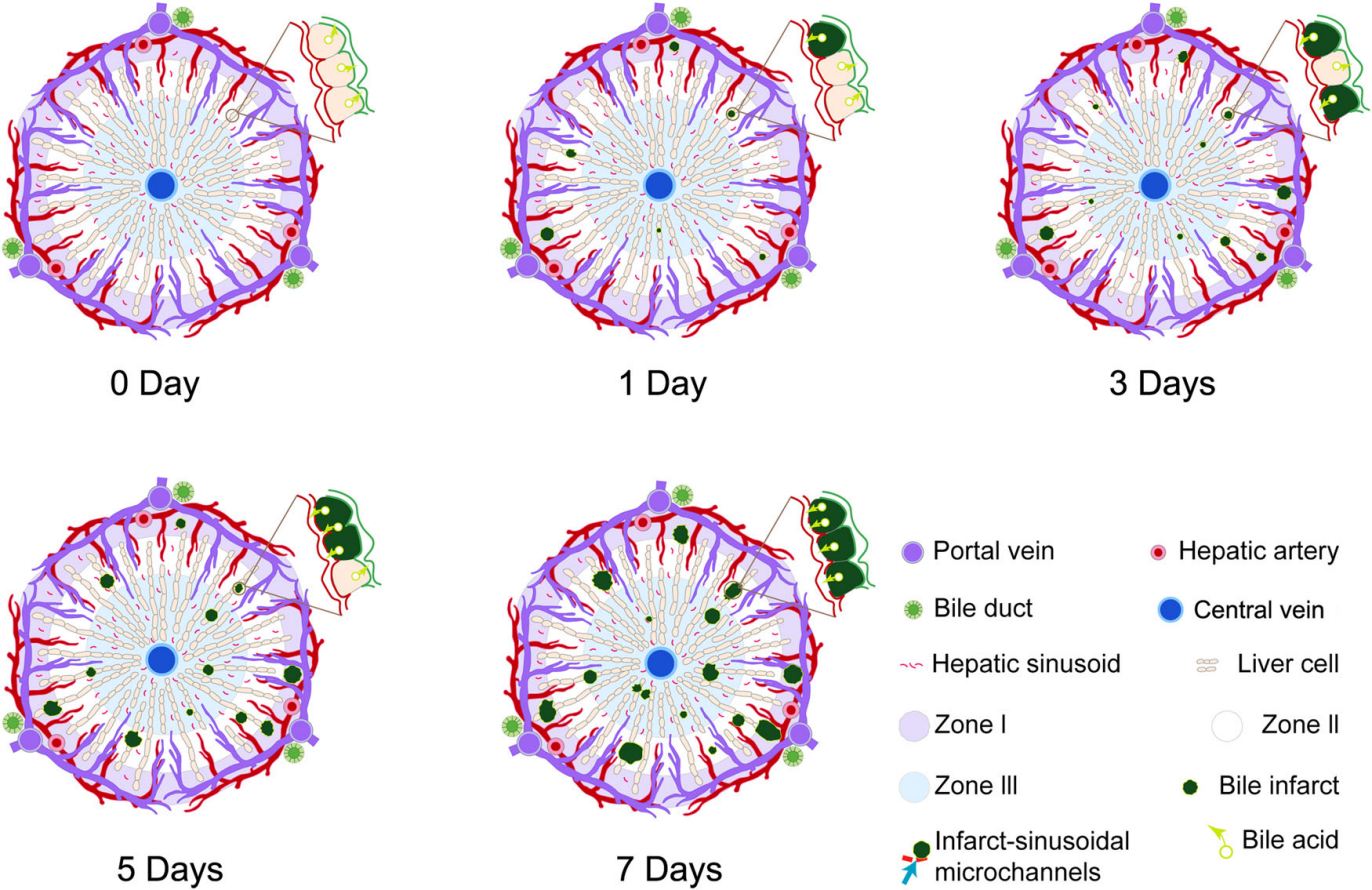
Schematic diagram of the evolution of the bile infarct in liver acini of the classic hepatic lobules (hexagonal structure). After BDL, the infarcts occur from the 1st day of BDL, and their volume and number increase remarkably with time after BDL. Moreover, the infarcts develop from zone I to zone III in the 3D acinus model during the disease progression. The bile infarct-sinusoidal microchannels emerge with the infarcts, and their diameter up to 3.5 µm means bile acids and other toxic compounds could directly leak from the infarcts into the hepatic sinusoids. Additionally, the microchannels gradually spread throughout the liver as the infarcts evolve in the progression of the disease. The diagram was created using Adobe Illustrator 2020 (Adobe, San Jose, CA).

This study comprehensively elucidates the evolution of bile infarcts during the course of obstructive biliary disease employing multiscale X-ray phase-contrast CT technology. The findings demonstrated that the phase-contrast CT could resolve the 3D structure and spatial distribution of the infarcts, portal veins and the hepatic venous system in the intact liver lobe at the macroscopic level and depict individual infarcts with the surrounding hepatic sinusoidal network at the microscopic level, confirmed by the histology. The 3D data presents a detailed scenario of how the bile infarct develops (Fig. 7). On the first day after BDL, the infarct begins to appear and then gradually increases in size, growing as an individual or merging with the neighboring infarcts. As the infarct grows, it continues to erode the sinusoidal space of the liver and forms an infarct-sinusoidal microchannel with the hepatic sinusoids. To explore distribution pattern of the infarct, we proposed a 3D model of hepatic acinus and found that the infarct starts from zone I, then gradually develops to zone II and finally concentrates in zone III during the disease process. Meanwhile, the infarct-sinusoidal microchannels spread throughout the liver as the infarct develops. Phase-contrast CT therefore captured the morphological alterations of the infarcts on a 3D level, which might give more insight about the disease procession as compared to conventional 2D histology^7^ or fluorescence microscopy^12^ on a comparable scale.

Elevated levels of serum bile acids are a primary characteristic of biliary obstruction in humans^22,23^ and rodents^24^, but the cause is controversial. Several routes of leakage of bile acids from the bile system into the blood have been reported: cholehepatic shunting^25^, vesicular regurgitation of bile acids from bile canaliculi through intact hepatocytes to sinusoids, and leakage through tight junctions^26–28^. A seminal study published recently has demonstrated that apical membrane rupture is the primary cause of bile leakage and that the subsequent formation of infarct-sinusoidal shunts plays a pivotal role in the transport of bile into the bloodstream^6^. The study suggests a new mechanism for the bile leakage, but the only regret is that it does not visualize the 3D spatial architecture of the shunts, nor their alterations following the formation. Utilizing phase-contrast CT, our study successfully imaged the shunt as a microchannel connecting the infarct and hepatic sinusoids at the submicron scale, allowing for both morphological and quantitative analysis. This microchannel was first observed on day 1 post-BDL, with an average of one per infarct, and subsequently increased in number, reaching a value of five on day 7. In addition to the growing number of microchannels possessed by the individual infarct, the number of the infarcts also grows, with microchannels spreading throughout the liver as the disease progresses. The diameter of the microchannel was approximately 3 µm, which allows toxic substances such as bile acids to pass directly through. Our findings further confirm the key role played by bile infarcts in bile leakage, and the exhibition of this microchannel provides additional evidence for this mechanism proposed by the previous study^6^.

Due to the lack of suitable imaging tools, the evolution of infarct in the intact liver remains poorly understood. 3D mapping of the infarct using phase-contrast CT offers the possibility of solving this problem ex vivo. The findings showed that the number and total volume of infarcts in intact liver lobes and their volume proportion in the whole lobes showed a remarkable growth trend with the time after BDL, indicating that the infarcts provided more distribution volume for the bile acids in the later period after BDL. To further analyze the evolution of the infarct, we proposed a 3D hepatic acinar model based on 2D hepatic acinar theory, which divides the whole liver into three equal parts with different oxygenation and metabolism^29^. After BDL, infarcts first appeared in zone I, which is consistent with previous reports^6,30^, and the number of infarcts in this zone accounted for approximately half of the total (day 1 post-BDL). As the disease progressed, the situation began to reverse, with infarcts in the zones II and III growing more rapidly, especially in zone III, where the number of infarcts in this region was about half the total number by day 7 post-BDL. We speculate that the characteristics of the hepatocytes within the different zones may be the cause of this phenomenon. The bile secretion function of hepatocytes is maximal in zone I^31^, where the apical membrane is under the greatest pressure after BDL and therefore the first to rupture and finally form an infarct. The flow of bile into the blood through the infarcts in zone I would affect the hepatocytes in zones II and III, which are vulnerable to toxic substances in the blood, especially in zone III^32^, so it is possible that the infarcts in these two zones are the result of high concentrations of toxic substances such as bile acids in the blood. This speculation offers a possible explanation for the distribution changes of infarcts in the three zones, but further experimental confirmation is needed. In brief, the evolution of infarct is like a double-edged sword. Their volume, number, morphology and overall distribution within the liver facilitate bile storage and biliary pressure partitioning. However, at the same time, the infarcts gradually erode the living space of the liver parenchyma, contributing to the progression of the disease.

In addition, the growth characteristics of individual infarcts were also explored on the CT images. The infarcts grew in two forms: individually and confluently. The volume of individual infarcts implied that the infarcts predominantly grow separately in the early stages, and as the disease progressed, the infarcts began to fuse to form large infarcts and became irregular in shape, consistent with the sphericity data. The initial effect of bile toxicity on hepatocytes was mild, with infarcts approaching a spherical shape; later the effect became progressively stronger and the shape tended to be disorganized. The growth characteristics of the infarcts provided by phase-contrast CT complement existing mechanisms of the infarct formation, while morphological changes in individual infarcts are consistent with the disease progression, providing a potentially valuable indicator for predicting the disease progression.

This study has two main limitations. A limitation of the present study is the use of ex vivo liver specimens. Phase-contrast CT offers outstanding advantages in 3D imaging of soft tissues and depth of detection, but has shortcomings in live and real-time imaging. Although some attempts have been made to use phase-contrast CT for in vivo studies^33,34^, real-time dynamic imaging of the liver is still a long way off. Another limitation is that although BDL is an experimental procedure that mimics obstructive biliary disease in humans^6^, this does not mean that the events in the model are exactly consistent with those in humans. Therefore, the sequence of events in the BDL model need to be further verified in human data.

In conclusion, by exploiting the excellent soft-tissue microscopic imaging capabilities of phase-contrast CT, our study provides a comprehensive analysis of the evolutionary features of the bile infarct in obstructive biliary disease. The presentation of the infarct-sinusoidal microchannels and the distribution pattern of the infarcts in the 3D hepatic acinus offers a new perspective for understanding the progression of obstructive biliary disease.

## Methods

### Ethics statement

All animal experiments were performed in accordance with the Guide for the Care and Use of Laboratory Animals approved by the Research Ethics Committee of the Beijing Friendship Hospital, Capital Medical University. We have complied with all relevant ethical regulations for animal use.

### Sample preparation

Male wild-type C57BL/6 mice (8–10 weeks old, weighing 18–20 g) were used and housed in a constant temperature environment with a 12-h light-dark cycle and free access to water and food. All animals were randomly divided into five groups: the control group and groups 1, 3, 5, and 7 days after BDL (*n* = 6 for each group). BDL was performed to induce biliary obstruction, which is a frequently applied experimental procedure with the aim to mimic human cholestatic disease in experimental animals and to gain insight into disease mechanisms^21^. Briefly, the mouse was anesthetized using phenobarbitone, and then its legs were fixed using tape to expose the abdomen. An incision was made on the abdominal midline. The liver was lifted toward the head, and the intestine was moved conversely. The common bile duct was isolated from the hepatic artery and portal vein. Double ligations were performed on the common bile duct using 5-0 silk and the bile duct was cut between the two ligations to simulate complete biliary obstruction in humans. Afterward, the liver and intestine were moved back to their normal positions. The abdomen was sutured. Subsequently, the mice were housed in cages and kept warm until they recovered and woke up. After the BDL surgery, the mice were sacrificed on days 1, 3, 5, and 7. They were perfused with 0.9% NaCl solution through the left ventricle, and the livers were harvested. Meanwhile, the common bile duct was exposed without ligation in the control group. All livers were fixed in 10% neutral buffered formalin solution for subsequent experiments. Before image acquisition, the liver samples were dehydrated with a graded series of ethanol solutions (50%, 70%, 80%, and 95% for 2 h each and 100% ethanol for 24 h).

### Principle of phase-contrast CT

The behavior of the X-ray beam while traversing a sample can be defined by the 3D complex refractive index distribution, $n\left(x,y,z\right)=1-\delta \left(x,y,z\right)+i\beta (x,y,z)$, where the real part $\delta$ represents the refractive index decrement, which describes the X-ray phase-shift; the imaginary part $\beta$ denotes the attenuation index responsible for the X-ray attenuation; and $(x,y,z)$ are the spatial coordinates^35^. Since Röntgen discovered X-rays in 1895, X-ray attenuation information $(\beta )$ has been widely utilized in medical imaging, but its limitations are also obvious and it is not suitable for biological soft tissue imaging. As opposed to conventional X-ray absorption imaging, which relies solely on the attenuation of the X-rays in matter, phase-contrast imaging is sensitive to X-ray phase shifts $(\delta )$. Early studies have reported that $\delta$ can be up to three orders of magnitude larger than $\beta$ for soft tissues^36,37^. Additionally, enhanced coherence properties and higher photon fluxes of third-generation synchrotrons have promoted phase-contrast imaging techniques for the investigation of weakly absorbing biological structures at the micron or even submicron level. As a combination of phase-contrast imaging and CT, phase-contrast CT can achieve high-resolution 3D imaging of biological soft tissues on the micron scale.

### CT scan

**Fig. 8:**
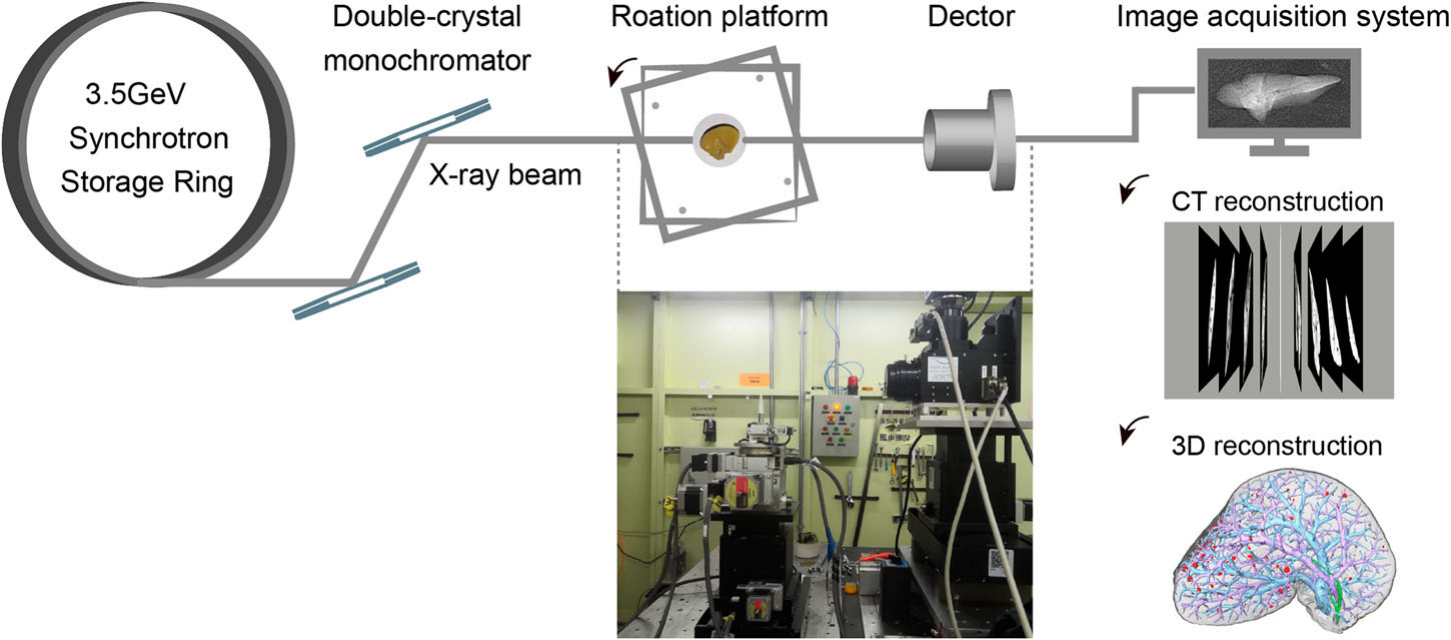
Schematic illustrating the imaging procedure and experimental setup of beamline BL13HB at the Shanghai Synchrotron Radiation Facility. In this experimental setup, parallel X-rays are derived from a 3.5-GeV electron storage ring and are monochromatized by a double silicon 111-crystal system. The monochromatic synchrotron beam is projected onto a sample fixed on a rotation platform, which can be rotated 180° to acquire the projection images of the sample at different angles; then, the transmitted beam is recorded by an image detector and displayed by the image acquisition system. Finally, the stack of CT sections and the 3D reconstruction of liver tissues are obtained through image postprocessing. Except for the photograph of the line station and the collected images of the sample, all other image elements were generated by Microsoft PowerPoint.

Phase-contrast CT imaging of liver samples was performed at the BL13HB beamline experimental station of Shanghai Synchrotron Radiation Facility (SSRF) in China. The experimental station includes a 3.5-GeV storage ring, a double-crystal monochromator, a sample stage, a CCD camera (pco.2000, PCO AG, Kelheim, Germany) coupled with optical lens, and an image acquisition system. A schematic of the experimental setup and imaging procedure are described in Fig. 8. Further details of the imaging system and the beamline are provided on the website (http://e-ssrf.sari.ac.cn/beamlines/bl13w1/) and in a related reference^38^. Two-pixel sizes (3.25 and 0.65 µm) were used, and the parameters used during image acquisition are listed in Table 1. The sample-to-detector distance was determined by a combination of X-ray energy, the principle of phase contrast imaging, and the minimum resolvable tissue size inside the sample (e.g., the diameter of the smallest resolvable hepatic sinusoid), as detailed in the Supplementary Methods of the Supplementary Information. The intact liver lobes were imaged with 3.25 µm pixel size. When CT imaging is performed at the 0.65 µm pixel size, smaller parts would be excised from these intact lobes to accommodate the reduced field of view. The flat-field images were recorded with the sample out of the field of view, and the dark-field images were obtained with no X-ray photons. After CT scan was completed, the samples were placed in formalin solution waiting for pathology experiments.

**Table 1:** Experimental parameters of the phase-contrast CT setup at two-pixel sizes

Experimental parameter	3.25-μm pixel size	0.65-μm pixel size
Photon energy (keV)	16	14
Field of view (mm)	6.656 (horizontal) × 5(vertical)	1.331 (horizontal) × 1.331 (vertical)
Matrix size (pixels)	2048 × 2048	2300 × 2300
Sample-to-detector distance (cm)	28	16
Projection images	1200	900
Exposure time per projection (ms)	100	100
CT scanning time per specimen (min)	15	18
of flat-field images	10	8
of dark-field images	5	5

### CT and 3D reconstruction

After image acquisition, flat-field and dark-field corrections were conducted on the raw projections to modulate the intensity profile caused by the detector and beam inhomogeneities. Phase retrieval was subsequently performed to extract the phase information using a phase-attenuation duality Born-type approximation algorithm^39^. A series of CT slices were reconstructed from the processed projections using the filtered back-projection algorithm. These procedures were implemented in PITRE (applied at the BL13HB station, SSRF). 3D visualization and image analysis were performed using the software Amira (version 2019.1, Thermo Fisher Scientific, American). CT stacks were imported to the Amira, and volume rendering and surface reconstruction were performed to visualize the liver. The hepatic vein, portal vein, bile duct, and bile infarcts were segmented and labeled within the segmentation module. The vessels were segmented using a region-growing operation, and the bile infarcts were segmented using threshold segmentation in 3D space. The vessels were segmented using a region-growing operation, and the bile infarcts were segmented in 3D space using threshold segmentation as they had a lower density and appeared with darker gray level than the surrounding healthy tissues. After binarization, the surface of the hepatic vein, portal vein, bile duct, and bile infarcts were generated and marked with different colors.

### Imaging analysis

Based on phase-contrast CT technology and 3D reconstruction, high-definition 3D virtual histological information of the intrahepatic structures could be obtained. To analyze the evolution of the infarct after BDL in detail, we divided the liver into different partitions according to its functional characteristics, and quantitatively evaluated the distribution and morphologic parameters of the infarcts in these partitions.

To identify bile infarcts in the whole liver-hepatic vein, portal vein, and bile infarcts in intact livers at different time points after BDL were presented in 3D form. Subsequently, parameters such as the total number and volume of infarcts in each hepatic lobe, the volume and sphericity of a single infarct, and the volume ratio of the bile infarct in one liver lobe were accurately evaluated, which could be obtained directly through the corresponding modules of the Amira software. The sphericity of the bile infarct ($\Psi$) was defined as:

$$\Psi =\frac{{\pi }^{1/3}{(6V)}^{2/3}}{A},$$

where $A$ represents the surface of one bile infarct and $V$ represents the volume of one bile infarct.

**Fig. 9:**
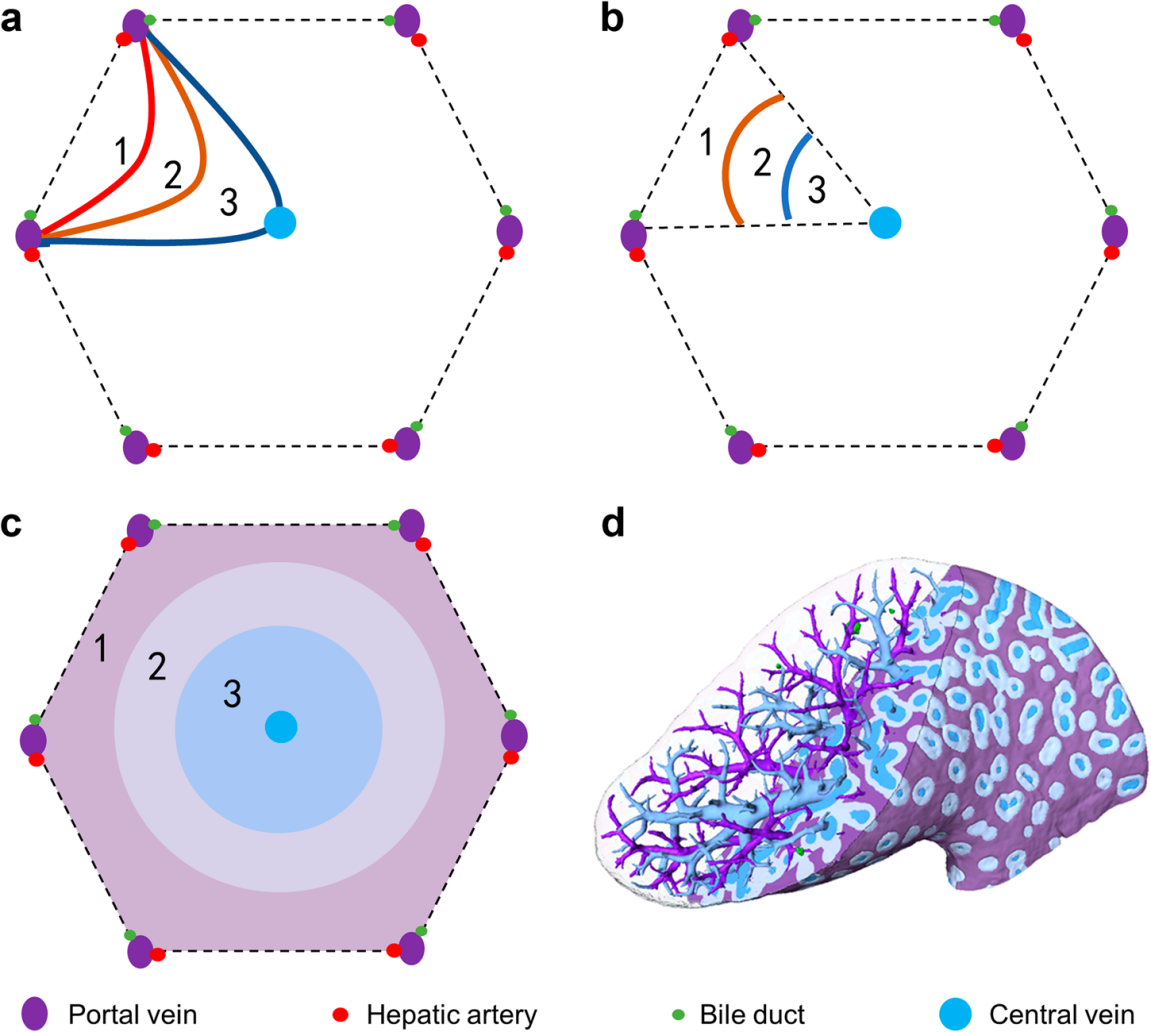
A schematic diagram of the interpretation and application of 3D hepatic acinus. **a** The classic hepatic acinus in a classic hepatic lobule which is a hexagonal structure centered on the central vein with the portal area (including the portal vein, hepatic artery, and bile duct) at its apex. **b** The proposed hepatic acinus model in the hepatic lobule. The model takes the central vein as the center and expand it to generate zone III, and then continues to expand on the basis of zone III to generate zone II. The remaining area around the portal vein is labeled zone I. During the process, the parameters need to be adjusted to make the volumes of the three zones equal. **c** Expansion of the hepatic acinus model to the whole hepatic lobule. **d** 3D exhibition of proposed hepatic acinus model (zone I in purple, zone II in white, and zone III in blue), together with the central veins (dark blue), portal veins (dark purple).

To identify bile infarcts in 3D hepatic acinus-The hepatic acinus is a functional unit of the liver parenchyma and has no distinct morphologic boundaries. It was first proposed by ref. ^40^, in which a portal tract is the axis, and the peripheral boundary is circumscribed by an imaginary line connecting the adjacent terminal hepatic venules (central hepatic venules of the classic lobule). The classical hepatic acinus model divides the hepatic parenchyma (liver cells) into three functional zones according to the direction of blood flow (i.e., the order in which the cells receive their blood supply) and mainly describes the differences in the ability of hepatocytes to obtain oxygen and nutrients within the different zones. Zone I: the area close to the portal vein, where hepatocytes are preferentially supplied with oxygen-rich nutrients and have an active cellular metabolism. Zone II: between Zone I and III, the nutritional condition of the hepatocytes is inferior to that of Zone I. Zone III: the area near the central vein, which is the area furthest from the incoming blood vessels, the marginal area of the hepatic acinus. Hepatocytes in this zone have poor nutritional conditions and weak cell regeneration capacity, and are susceptible to damage from drugs and toxic substances. However, the hepatic acinus in two dimensions was not suitable for analyzing the spatial distribution of the 3D bile infarcts. In this study, a 3D liver acinus model based on phase contrast CT images is proposed in combination with the obtained 3D image data and the definition of the liver acinus and hepatic lobule (Fig. 9). The first step was to take the hepatic/central vein as the center and expand it based on the sphere dilation algorithm to generate zone III (excluding the hepatic/central vein). The second step is to continue to expand on the basis of zone III to generate zone II. In step 3, the remaining area around the portal vein is labeled zone I (excluding the portal vein). During the process, the parameters of the sphere dilation algorithm need to be adjusted to make the volumes of the three zones equal and each of the zones is morphologically influenced by the spatial structure of the portal and hepatic/central veins. This algorithm can generate a 3D hepatic acinar model directly from the 3D data. If the infarct spans two or even three zones, the zone with the largest proportion of the infarct volume will be used as its label. In this model, the effectiveness of the 3D hepatic vesicle depends on the accuracy of the segmentation of the vascular system (especially the terminal vessels). Two approaches were employed to ensure the accuracy of the segmentation. First, the vessels on the CT and pathology sections were matched and compared to ensure that the smallest vessels visible on the CT slices were accurate. Second, after acquiring the high-resolution data, we superimposed this data on the low-resolution data of the intact liver lobes to further validate the accuracy of the minimum level of vessel segmentation. Subsequently, the number, volume and sphericity of infarcts in each zone were measured and analyzed. The number fraction and total volume of the infarcts, and the volume and sphericity of a single infarct in each zone were measured and analyzed.

To identify bile infarct-sinusoid microchannel—the microscopic structure of hepatic sinusoids and the infarcts could be observed at the submicron level. Based on CT images at this scale, the microchannels between the infarcts and sinusoids were studied, and 3D segmentation of the microchannel was carried out. Then, the number of microchannels of a single infarct at different time points after BDL was measured and analyzed.

To observe the growth characteristics of the infarct—the pattern of infarct growth can be observed at a 0.65 µm pixel size. The infarcts can be classified into two types: one is the continuous expansion of an individual infarct area, that is, the individual infarct without fusion, and the other is the fusion between multiple infarcted areas, that is, the confluent infarct. Then, the volume of individual and confluent infarcts was quantified, and the proportion of both in the liver was analyzed in each group.

### Histological analysis

After phase-contrast CT scans, the livers were dehydrated and embedded in paraffin. Sections with a slice thickness of 4 μm were stained with hematoxylin and eosin (H&E). Pathological images were digitized via a dedicated camera. Coregistration of the phase-contrast CT images and the corresponding histopathologic findings was manually performed to confirm the accuracy of the phase-contrast CT technique (Fig. 1).

### Statistics and reproducibility

The assumption of normality was evaluated using the Shapiro‒Wilk test. The normalized results are presented as the mean ± standard deviation and were compared using independent sample *t* tests when assumptions of normality could be verified, such as with the number, total volume, volume ratio of the bile infarcts and liver lobe, and fraction of the number. The data are presented as the median (interquartile range, IQR) when assumptions of normality could not be verified, and the Mann‒Whitney *U* test was used for group comparisons, such as the volume of a single infarct and sphericity. The analyses were carried out using SPSS software (version 20, IBM, Chicago, USA). A *p* value < 0.05 was considered statistically significant.

### Reporting summary

Further information on research design is available in the Nature Portfolio Reporting Summary linked to this article.

### Supplementary information

Supplementary Information

Description of Additional Supplementary Files

Supplementary Data 1

Supplementary Movie S1

Supplementary Movie S2

Supplementary Movie S3

Reporting Summary

## Data Availability

The numerical source data for figures and plots can be found in Supplementary data 1. All other data are available from the corresponding author upon reasonable request.
