# Supplementary material for: Multiscale X-ray phase-contrast CT unveils the evolution of bile infarct in obstructive biliary disease
Source: Commun Biol. 2024 Apr 23;7:490. doi: 10.1038/s42003-024-06185-7 (PMC11039475; doi:10.1038/s42003-024-06185-7)
Supplement: Supplementary file 1 — Supplementary Information [file 42003_2024_6185_MOESM1_ESM.pdf]

Supplementary information for:

Multiscale X-ray Phase-contrast CT Unveils the Evolution of Bile Infarct in Obstructive Biliary Disease

Xiaohong Xin[1#], Jianbo Jian[2#], Xu Fan[3,4#], Beining Qi[1], Yuanyuan Zhao[1], Wenjuan Lv[1], Yuqing Zhao[1], Xinyan Zhao[3,4], Chunhong Hu[1]

[1] School of Biomedical Engineering and Technology, Tianjin Medical University, Tianjin 300070, China

[2] Department of Radiation Oncology, Tianjin Medical University General Hospital, Tianjin 300052, China

[3] Liver Research Center, Beijing Friendship Hospital, Capital Medical University, Beijing100050, China

[4] Beijing Key Laboratory of Translational Medicine on Liver Cirrhosis and National Clinical Research Center of Digestive Disease, Beijing100050, China

[#] Xiaohong Xin, Jianbo Jian and Xu Fan contributed equally to this work.

Corresponding to:

Chunhong Hu PhD.

E-mail：chunhong_hu@hotmail.com

Co-correspondence to:

Xinyan Zhao PhD.

E-mail: zhao_xinyan@ccmu.edu.cn

Supplementary Methods

Sample-to-detector distance

In this study, the distance z between the sample and the detector plane fulfils the near-field condition, i.e.

$$z \ll d^2/\lambda ,$$

where d is the characteristic size of the smallest discernible features in the sample, and λ is the X-ray wavelength. We have explored the size of the smallest resolvable structures on CT images of different pixel sizes. At the 3.25 μm pixel size, the diameter of the smallest resolvable blood vessel is 13 μm and the energy is 16 keV, a condition that gives a d^2/λ of about 2 m. At the 0.65 μm pixel size, the diameter of the smallest resolvable hepatic sinusoid is 3.25 μm and the energy is 14 keV, a condition that yields a d^2/λ of about 12 cm. Prior to the CT scan, we evaluated the image quality at different distances and finally chose the sample-to-detector distances of 28 cm at the 3.25 μm and 16cm at the 0.65 μm. According to the previous formula, the actual value of the sample-to-detector distance value at 0.65 μm pixel size is a little larger than the theoretical value. This is because the quality of the images we measured was not particularly good at the value of the sample-to-detector distance less than 12 cm, and at 16 cm the image quality met our expectations and could clearly reveal the spatial structure of the hepatic sinusoids and infarcts, and was consistent with the pathological findings, confirming the accuracy of the CT images. We speculate that this may be due to the fact that microstructures such as hepatic sinusoids in liver tissue are poorly differentiated from the surrounding tissues, and the sample-to-detection distance needs to be lengthened to enhance the resolving power of

the image. Thus, the choice of detector distances at different pixel sizes integrates factors such as the characteristics of the sample tissue composition and x-ray energy, which requires a balance between imaging parameters, and ultimately achieves the imaging resolution that meets the requirements of our study of the evolution of bile infarcts and is the optimal choice under the influence of various factors.
