# Supplementary material for: Multiscale X-ray phase-contrast CT unveils the evolution of bile infarct in obstructive biliary disease
Source: Commun Biol. 2024 Apr 23;7:490. doi: 10.1038/s42003-024-06185-7 (PMC11039475; doi:10.1038/s42003-024-06185-7)
Supplement: Supplementary file 2 — Description of Additional Supplementary Files [file 42003_2024_6185_MOESM2_ESM.pdf]

Description of Additional Supplementary Files

File Name: Supplementary Data 1

Description: The numerical source data for figures and plots.

File Name: Supplementary Movie S1

Description: The division of the 3D liver acinus. The central vein is pseudocolored in blue, the portal vein in purple, zone III in blue, zone II in white, and zone I in purple.

File Name: Supplementary Movie S2

Description: The endoscopic view of the bile infarct in the 3D liver acinus. The bile infarct is pseudocolored in red, zone III in blue, zone II in white, and zone I in purple.

File Name: Supplementary Movie S3

Description: The bile infarct surrounded by the sinusoid and an endoscopic view of the connection between the bile infarct and the hepatic vein through the sinusoid. The central vein is pseudocolored in blue, the portal vein in purple, the sinusoid in pink, and the bile infarct in red.
