# Supplementary material for: Multiscale X-ray phase-contrast CT unveils the evolution of bile infarct in obstructive biliary disease
Source: Commun Biol. 2024 Apr 23;7:490. doi: 10.1038/s42003-024-06185-7 (PMC11039475; doi:10.1038/s42003-024-06185-7)
Supplement: Supplementary file 7 — Reporting Summary [file 42003_2024_6185_MOESM7_ESM.pdf]

nature portfolio

Corresponding author(s): Chunhong Hu

Last updated by author(s): Apr 4, 2024

Reporting Summary

Nature Portfolio wishes to improve the reproducibility of the work that we publish. This form provides structure for consistency and transparency in reporting. For further information on Nature Portfolio policies, see our Editorial Policies and the Editorial Policy Checklist.

Statistics

For all statistical analyses, confirm that the following items are present in the figure legend, table legend, main text, or Methods section.

n/a	Confirmed	
☐	☒	The exact sample size (n) for each experimental group/condition, given as a discrete number and unit of measurement
☐	☒	A statement on whether measurements were taken from distinct samples or whether the same sample was measured repeatedly
☐	☒	The statistical test(s) used AND whether they are one- or two-sided<br>*Only common tests should be described solely by name; describe more complex techniques in the Methods section.*
☒	☐	A description of all covariates tested
☐	☒	A description of any assumptions or corrections, such as tests of normality and adjustment for multiple comparisons
☐	☒	A full description of the statistical parameters including central tendency (e.g. means) or other basic estimates (e.g. regression coefficient) AND variation (e.g. standard deviation) or associated estimates of uncertainty (e.g. confidence intervals)
☐	☒	For null hypothesis testing, the test statistic (e.g. F, t, r) with confidence intervals, effect sizes, degrees of freedom and P value noted<br>*Give P values as exact values whenever suitable.*
☒	☐	For Bayesian analysis, information on the choice of priors and Markov chain Monte Carlo settings
☒	☐	For hierarchical and complex designs, identification of the appropriate level for tests and full reporting of outcomes
☒	☐	Estimates of effect sizes (e.g. Cohen's d, Pearson's r), indicating how they were calculated

Our web collection on statistics for biologists contains articles on many of the points above.

Software and code

Policy information about availability of computer code

Data collection	No software was used.
Data analysis	A series of CT slices were reconstructed from the processed projections using the filtered back-projection algorithm. These procedures were implemented in PITRE (applied at the BL13HB station, SSRF). 3D visualization and image analysis were performed using the software Amira (version 2019.1, Thermo Fisher Scientific, American).

For manuscripts utilizing custom algorithms or software that are central to the research but not yet described in published literature, software must be made available to editors and reviewers. We strongly encourage code deposition in a community repository (e.g. GitHub). See the Nature Portfolio guidelines for submitting code & software for further information.

Data

Policy information about availability of data

All manuscripts must include a data availability statement. This statement should provide the following information, where applicable:

- Accession codes, unique identifiers, or web links for publicly available datasets
- A description of any restrictions on data availability
- For clinical datasets or third party data, please ensure that the statement adheres to our policy

The numerical source data for figures and plots can be found in Supplementary data 1. All other data are available from the corresponding author on reasonable request.

Research involving human participants, their data, or biological material

Policy information about studies with human participants or human data. See also policy information about sex, gender (identity/presentation), and sexual orientation and race, ethnicity and racism.

Reporting on sex and gender	n.a. i
Reporting on race, ethnicity, or other socially relevant groupings	n.a. i
Population characteristics	n.a. i
Recruitment	n.a. i
Ethics oversight	n.a. i

Note that full information on the approval of the study protocol must also be provided in the manuscript.

Field-specific reporting

Please select the one below that is the best fit for your research. If you are not sure, read the appropriate sections before making your selection.

☒ Life sciences ☐ Behavioural & social sciences ☐ Ecological, evolutionary & environmental sciences

For a reference copy of the document with all sections, see nature.com/documents/nr-reporting-summary-flat.pdf

Life sciences study design

All studies must disclose on these points even when the disclosure is negative.

Sample size	The study ensured that there were more than five animals in each group to guarantee that our subsequent measurements were statistically significant.
Data exclusions	No data were excluded from the analyses.
Replication	All measurements are performed based on software platforms such as Amira to ensure the objectivity and reproducibility of the data.
Randomization	All animals were randomly divided into 5 groups: the control group and Groups 1, 3, 5, and 7 days after BDL (n=6 for each group).
Blinding	The investigators were blinded to group allocation during data collection and analysis.

Reporting for specific materials, systems and methods

We require information from authors about some types of materials, experimental systems and methods used in many studies. Here, indicate whether each material, system or method listed is relevant to your study. If you are not sure if a list item applies to your research, read the appropriate section before selecting a response.

Materials & experimental systems

n/a	Involved in the study	
☒	☐	Antibodies
☒	☐	Eukaryotic cell lines
☒	☐	Palaeontology and archaeology
☐	☒	Animals and other organisms
☒	☐	Clinical data
☒	☐	Dual use research of concern
☒	☐	Plants

Methods

n/a	Involved in the study	
☒	☐	ChIP-seq
☒	☐	Flow cytometry
☒	☐	MRI-based neuroimaging

Animals and other research organisms

Policy information about studies involving animals; ARRIVE guidelines recommended for reporting animal research, and Sex and Gender in Research

Laboratory animals	Male C57BL/6 mice (8-10 weeks old, weighing 18-20 g) were used.
Wild animals	The study did not involve wild animals.
Reporting on sex	Male animals were selected for this study and have also been selected for most other bile duct ligation (BDL) modeling studies.
Field-collected samples	The study did not involve samples collected from the field.
Ethics oversight	All animal experiments were performed in accordance with the Guide for the Care and Use of Laboratory Animals approved by the Research Ethics Committee of the Beijing Friendship Hospital, Capital Medical University. We have complied with all relevant ethical regulations for animal use.

Note that full information on the approval of the study protocol must also be provided in the manuscript.

Plants

Seed stocks	n.a. i
Novel plant genotypes	n.a. i
Authentication	n.a. i
